# Asymptotic Normality for Plug-In Estimators of Generalized Shannon’s Entropy

**DOI:** 10.3390/e24050683

**Published:** 2022-05-12

**Authors:** Jialin Zhang, Jingyi Shi

**Affiliations:** Department of Mathematics and Statistics, Mississippi State University, Mississippi State, MS 39762, USA; jshi@math.msstate.edu

**Keywords:** Shannon’s entropy, generalized Shannon’s entropy, plug-in estimation, asymptotic normality

## Abstract

Shannon’s entropy is one of the building blocks of information theory and an essential aspect of Machine Learning (ML) methods (e.g., Random Forests). Yet, it is only finitely defined for distributions with fast decaying tails on a countable alphabet. The unboundedness of Shannon’s entropy over the general class of all distributions on an alphabet prevents its potential utility from being fully realized. To fill the void in the foundation of information theory, Zhang (2020) proposed generalized Shannon’s entropy, which is finitely defined everywhere. The plug-in estimator, adopted in almost all entropy-based ML method packages, is one of the most popular approaches to estimating Shannon’s entropy. The asymptotic distribution for Shannon’s entropy’s plug-in estimator was well studied in the existing literature. This paper studies the asymptotic properties for the plug-in estimator of generalized Shannon’s entropy on countable alphabets. The developed asymptotic properties require no assumptions on the original distribution. The proposed asymptotic properties allow for interval estimation and statistical tests with generalized Shannon’s entropy.

## 1. Introduction

### 1.1. Introduction and Related Work

Shannon’s entropy, introduced by [1], is one of the building blocks of Information Theory and a key aspect of Machine Learning (ML) methods (e.g., Random Forests). It is one of the most popular quantities on countable alphabet (An countable alphabet is a space that could be either finite, or countably infinite; the elements in an alphabet can be either ordinal (e.g., numbers) or non-ordinal (e.g., letters)), particularly on non-ordinal space with categorical data. For example, in [2], all reviewed feature selection methods on non-ordinal space boiled down to a function of Shannon’s entropy. In addition, Shannon’s entropy is one of the most important foundations for all tree-based ML algorithms, sometimes substitutable with the Gini impurity index [3,4,5].

As one of the essential information-theoretical quantities, Shannon’s entropy and its estimation are widely studied in the past decades [6,7,8,9,10,11,12]. In particular, [9] proved that an unbiased estimator of Shannon’s entropy does not exist. Current state-of-art Shannon’s entropy point estimator was provided in [10] with the fastest bias decaying rate (exponentially-decaying).

Nevertheless, Shannon’s entropy is only finitely defined for distributions with fast decaying tails [13].

It is never known if the real distribution yields a finite Shannon’s entropy in practice. Furthermore, all existing results on Shannon’s entropy require it to be finitely defined, which results in a usage restriction when adopting the entropy-based methods. This is, in fact, a void in the foundation of all Shannon’s entropy-related results.

**Example** **1** (Unbounded Shannon’s Entropy)**.** *Let a distribution P be $P_{k}=c/\left(k\ln^{2}k\right)$ for k≥2, where c is the constant that makes P a valid probability distribution. Such c uniquely exists because $\sum_{k=2}^{\infty }\left[1/\left(k\ln^{2}k\right)\right]$ converges. Then Shannon’s entropy of P, H(P) is unbounded because*

$$\begin{array}{cc} H(P)= & -\sum_{k=2}^{\infty }\left[P_{k}\ln P_{k}\right]\\ = & -\sum_{k=2}^{\infty }\left[\frac{c}{k\ln^{2}k}\ln\frac{c}{k\ln^{2}k}\right]\\ = & -\sum_{k=2}^{\infty }\left[\frac{c}{k\ln^{2}k}\ln c\right]+\sum_{k=2}^{\infty }\left[\frac{c}{k\ln^{2}k}\ln k\right]+\sum_{k=2}^{\infty }\left[\frac{c}{k\ln^{2}k}\ln\left(\ln^{2}k\right)\right]\\ = & -\sum_{k=2}^{\infty }\left[\frac{c}{k\ln^{2}k}\ln c\right]+\sum_{k=2}^{\infty }\left[\frac{c}{k\ln k}\right]+\sum_{k=2}^{\infty }\left[\frac{2c\ln\ln k}{k\ln^{2}k}\right]\\ = & \;A\;\mathrm{Finite}\;\mathrm{Value}+\infty +A\;\mathrm{Finite}\;\mathrm{Value}=\infty . \end{array}$$

The effort to generalize Shannon’s entropy has been long and extensive in the existing literature. As summarized in [14], the main perspective in the generalization in the existing literature is based on axiomatic characterization of Shannon’s entropy [15,16]. For example, Refs. [17,18] are efforts with respect to the functional form, $H=\sum_{k\geq 1}h\left(p_{k}\right)$, under certain desirable axioms, h(p)=−plogp is uniquely determined up to a multiplicative constant; if the strong additivity axiom is relaxed to be one of the weaker versions, say α-additivity or composability, then h(p) may be of other forms, which give rise to Rényi’s entropy [19], and the Tsallis entropy [20]. However, all such generalization effort does not seem to lead to an information measure on a joint alphabet that would possess all the desirable properties of mutual information, which is supported by an argument via the Kullback–Leibler divergence [21]. Interested readers may refer to [14] for details.

To further address the deficiency of Shannon’s entropy [14] proposed generalized Shannon’s entropy (GSE) and showed that GSE enjoys all properties of a finite Shannon’s entropy. In addition, GSE is finitely defined on all distributions. Due to the advantages of GSE and the deficiency of Shannon’s entropy, the use of Shannon’s entropy should eventually be transited to GSE.

### 1.2. Summary and Contribution

To aid the transition, the estimation of GSE needs to be studied. In practice, the plug-in estimator is one of the most popular estimation approaches. For plug-in estimation of GSE, asymptotic properties are required for statistical tests and confidence intervals. This article studies the asymptotic properties for plug-in estimators of GSE.

As a summary of the article’s results, Theorem 1 and Corollary 1 provide asymptotic normality properties for the plug-in estimators of GSE for all orders (An explanation of the order is given in Definition 2) on countably infinite alphabet. Corollary 2 provides the asymptotic normality properties for the plug-in estimators of GSE for all orders on finite alphabet, except the underlying distribution being uniform (Under a uniform distribution, the estimation of GSE is reduced to an estimation of population size. Interested readers can read [22]). The presented asymptotic normality properties immediately allow interval estimation and hypothesis testing with plug-in estimators of GSE. The numerical results in Section 3 show that the developed asymptotic properties converge fast, especially when the order is 2.

The presented properties and performance of GSE plug-in estimators suggest that GSE’s use is full of promising potential. One may be concerned the construction of CDOTC (Defined in Definition 1) would bring additional estimation challenges to the already-difficult estimation of Shannon’s entropy. Yet, the convergence speed for GSE plug-in estimators is fast. To further unlock the potentials of GSE, additional estimation methods of GSE and asymptotic properties of functions of GSE (e.g., Generalized Mutual Information, also originated in [14]) shall be visited. This article’s results and proofs’ approaches provide a solid direction toward that end.

The rest of this paper is organized as follows. Section 2 formally states the problem and gives our main results. In Section 3, we provide a small-scale simulation study. In Section 4, we discuss the potential of GSE. Proofs are postponed to Section 5.

## 2. Main Results

Let *Z* be a random element on a countable alphabet $Z=\left\{z_{k};k\geq 1\right\}$ with an associated distribution $p=\left\{p_{k};k\geq 1\right\}$. Let the cardinality or support on Z be denoted $K=\sum_{k\geq 1}1\left[p_{k}>0\right]$, where 1[·] is the indicator function. *K* is possibly finite or infinite. Let P denote the family of all distributions on Z. Shannon’s entropy, *H*, is defined as

$$H=H\left(Z\right)=-\sum_{k\geq 1}p_{k}\ln p_{k}.$$

To state our main result, we need to state Definitions 1 and 2 given by [14], and Definition 3.

**Definition** **1** (Conditional Distribution of Total Collision (CDOTC))**.** *Given $Z=\left\{z_{k};k\geq 1\right\}$ and $p=\left\{p_{k}\right\}$, consider the experiment of drawing an identically and independently distributed (iid) sample of size m (m≥2). Let $C_{m}$ denote the event that all observations of the sample take on a same letter in Z, and let $C_{m}$ be referred to as the event of total collision. The conditional probability, given $C_{m}$, that the total collision occurs at letter $z_{k}$ is*

$$p_{m,k}=\frac{p_{k}^{m}}{\sum_{i\geq 1}p_{i}^{m}},$$

*where m≥2. $p_{m}=\left\{p_{m,k}\right\}$ is defined as the m-th order CDOTC.*

**Definition** **2** (Generalized Shannon’s Entropy (GSE))**.** *Given $Z=\left\{z_{k};k\geq 1\right\}$, $p=\left\{p_{k}\right\}$, and $p_{m}=\left\{p_{m,k}\right\}$, generalized Shannon’s entropy (GSE) is defined as*

$$H_{m}\left(Z\right)=-\sum_{k\geq 1}p_{m,k}\ln p_{m,k},$$

*where $p_{m,k}$ is defined in Definition 1, and m=2,3,… is the order of GSE. GSE with order m is referred to as the m-th order GSE.*

It is clear that $p_{m}$ is a probability distribution induced from $p=\left\{p_{k}\right\}$. Furthermore, for each *m*, m≥2, p and $p_{m}$ uniquely determined each other (Lemma 1 in [14]). To help understand Definitions 1 and 2, Examples 2 and 3 are provided as follows.

**Example** **2** (The 2nd order CDOTC)**.** *Given $Z=\left\{z_{k};k\geq 1\right\}$ and $p=\left\{p_{k}\right\}=\left\{6k^{-2}/\pi^{2};k=1,2,3,\ldots \right\}$, the 2nd order CDOTC is then defined as*

$$p_{2}=\left\{p_{2,k}\right\},$$

*where*

$$p_{2,k}=\frac{p_{k}^{2}}{\sum_{i\geq 1}p_{i}^{2}}=\frac{36k^{-4}/\pi^{4}}{\sum_{i\geq 1}\left[36i^{-4}/\pi^{4}\right]}=\frac{k^{-4}}{\sum_{i\geq 1}i^{-4}}$$

*for k=1,2,3,….*

**Example** **3** (The 2nd order GSE)**.** *Given $Z=\left\{z_{k};k\geq 1\right\}$, $p=\left\{p_{k}\right\}=\left\{6k^{-2}/\pi^{2};k=1,2,3,\ldots \right\}$, and $p_{2}=\left\{p_{2,k}\right\}=\left\{\frac{k^{-4}}{\sum_{i\geq 1}i^{-4}};k=1,2,\ldots \right\}$, the 2nd order GSE, $H_{2}\left(Z\right)$, is then defined as*

$$H_{2}\left(Z\right)=-\sum_{k\geq 1}p_{2,k}\ln p_{2,k},$$

*where $p_{2,k}$ is given in Example 2.*

The definition of the plug-in estimator of GSE is stated in Definition 3.

**Definition** **3** (Plug-in estimator of GSE)**.** *Let $Z_{1},Z_{2},\ldots ,Z_{n}$ be independent and identically distributed (iid) random variables taking values in $Z=\left\{z_{k};k\geq 1\right\}$ with distribution p. For each k=1,2,…, let $Y_{k}=\sum_{i=1}^{n}1\left[Z_{i}=z_{k}\right]$ be the sample count of observations in category $z_{k}$, and let ${\hat{p}}_{k}=Y_{k}/n$ be the sample proportion. The plug-in estimator for the m-th order GSE, ${\hat{H}}_{m}\left(Z\right)$, is defined as*

$$\begin{array}{cc} {\hat{H}}_{m}\left(Z\right)= & -\sum_{k\geq 1}\left[{\hat{p}}_{m,k}\ln{\hat{p}}_{m,k}\right]\\ = & -\sum_{k\geq 1}\left[\frac{{\hat{p}}_{k}^{m}}{\sum_{i\geq 1}{\hat{p}}_{i}^{m}}\ln\frac{{\hat{p}}_{k}^{m}}{\sum_{i\geq 1}{\hat{p}}_{i}^{m}}\right]. \end{array}$$

Our main results are stated in Theorem 1, Corollary 1 and 2.

**Theorem** **1.** *Let $p=\{p_{k}\}$ be a probability distribution supported by a countably infinite alphabet, without any further conditions,*

$$\sqrt{n}\left({\hat{H}}_{m}\left(Z\right)-H_{m}\left(Z\right)\right)\overset{d}{\to }N\left(0,\sigma_{m}^{2}\right),$$

*where*

$$\sigma_{m}^{2}=\sum_{k=1}^{\infty }{\left[\frac{m^{2}}{p_{k}}\left(p_{m,k}\ln p_{m,k}+p_{m,k}H_{m}\left(Z\right)\right)\right]}^{2}.$$

**Corollary** **1.** *Let $p=\{p_{k}\}$ be a probability distribution supported by a countably infinite alphabet, without any further conditions,*

$$\sqrt{n}\left(\frac{{\hat{H}}_{m}\left(Z\right)-H_{m}\left(Z\right)}{{\hat{\sigma }}_{m}}\right)\overset{d}{\to }N\left(0,1\right),$$

*where*

(1) $${\hat{\sigma }}_{m}^{2}=\sum_{k=1}^{\infty }{\left[\frac{m^{2}}{{\hat{p}}_{k}}\left({\hat{p}}_{m,k}\ln{\hat{p}}_{m,k}+{\hat{p}}_{m,k}{\hat{H}}_{m}\left(Z\right)\right)\right]}^{2}.$$

**Corollary** **2.** *Let $p=\{p_{k};k=1,2,\ldots ,K\}$ be a non-uniform probability distribution on a finite alphabet, without any further conditions,*

$$\sqrt{n}\left(\frac{{\hat{H}}_{m}\left(Z\right)-H_{m}\left(Z\right)}{{\hat{\sigma }}_{m}}\right)\overset{d}{\to }N\left(0,1\right),$$

*where*

$${\hat{\sigma }}_{m}^{2}=\sum_{k=1}^{K}{\left[\frac{m^{2}}{{\hat{p}}_{k}}\left({\hat{p}}_{m,k}\ln{\hat{p}}_{m,k}+{\hat{p}}_{m,k}{\hat{H}}_{m}\left(Z\right)\right)\right]}^{2}.$$

Corollary 2 is a special case of Theorem 1. All proofs are provided in Section 5.

## 3. Simulations

One of the main applications of our results is the ability to construct confidence intervals, and hence testing hypothesis. Specifically, Corollary 1 implies that an asymptotic (1−α)100% confidence interval for $H_{m}$ is given by

(2) $$\left({\hat{H}}_{m}-z_{\alpha /2}\frac{{\hat{\sigma }}_{m}}{\sqrt{n}},{\hat{H}}_{m}+z_{\alpha /2}\frac{{\hat{\sigma }}_{m}}{\sqrt{n}}\right),$$

where ${\hat{\sigma }}_{m}$ is given by (1) and $z_{\alpha /2}$ is a number such that $P\left(Z>z_{\alpha /2}\right)=\alpha /2$ and Z∼ N(0,1). In this section, we give a small scale simulation study to check the finite sample performance of this confidence interval.

We consider Zeta distribution

$$P\left(x=k\right)=\frac{1}{\zeta (s)}k^{-s},\;k\in \left\{1,2,\ldots \right\}$$

with s=1.5 and 2.5, where ζ(s) is the Riemann zeta function given by

$$\zeta \left(s\right)=\sum_{n=1}^{\infty }\frac{1}{n^{s}}.$$

**Figure 1:**
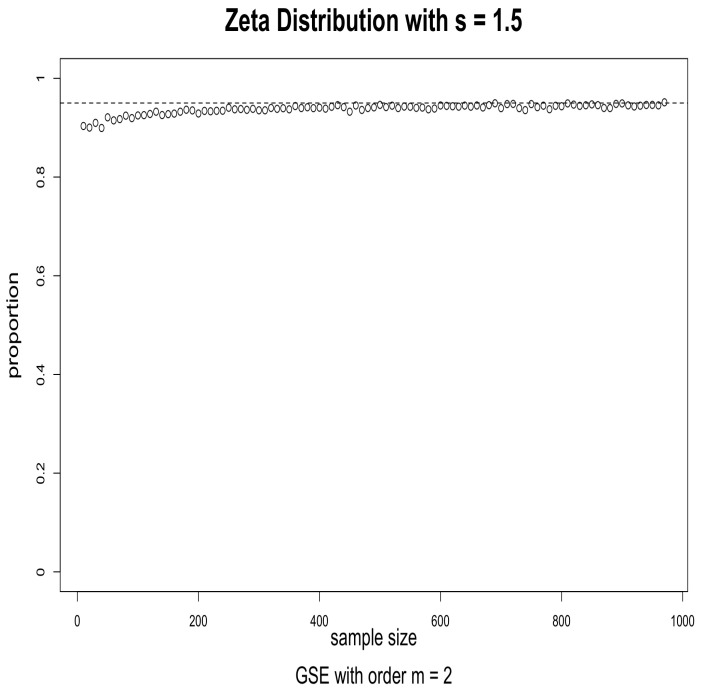
Effectiveness of the 95% confidence intervals as a function of sample size. Simulations from Zeta distribution with s=1.5 and GSE with order m=2. The horizontal dashed line is at 0.95.

**Figure 2:**
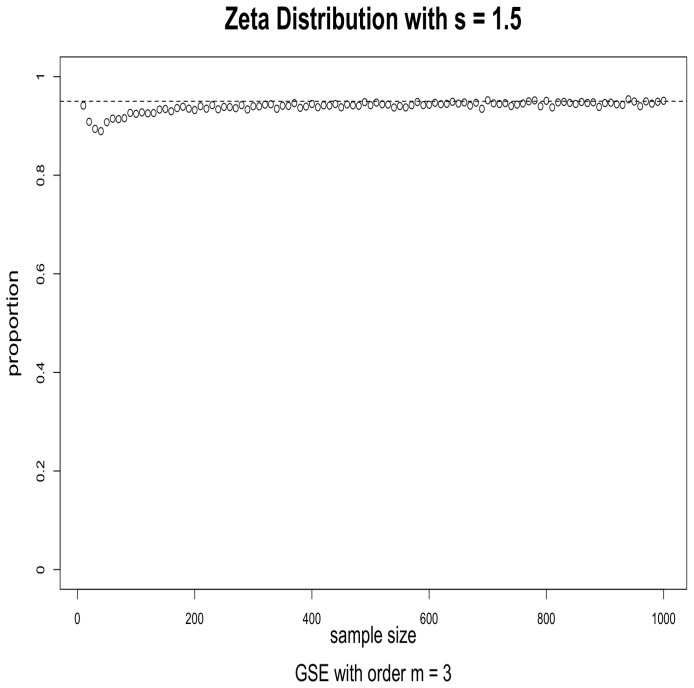
Effectiveness of the 95% confidence intervals as a function of sample size. Simulations from Zeta distribution with s=1.5 and GSE with order m=3. The horizontal dashed line is at 0.95.

**Figure 3:**
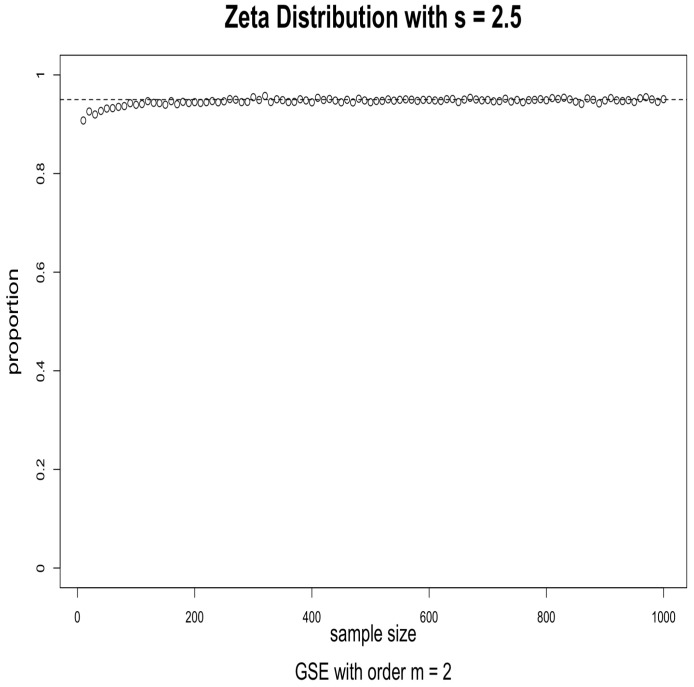
Effectiveness of the 95% confidence intervals as a function of sample size. Simulations from Zeta distribution with s=2.5 and GSE with order m=2. The horizontal dashed line is at 0.95.

**Figure 4:**
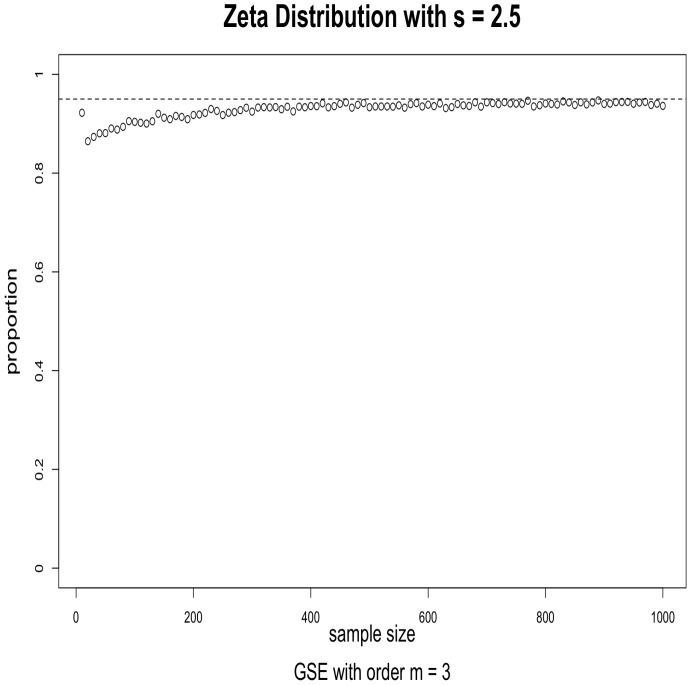
Effectiveness of the 95% confidence intervals as a function of sample size. Simulations from Zeta distribution with s=2.5 and GSE with order m=3. The horizontal dashed line is at 0.95.

The simulations were performed as follows. For the given distribution, we obtained a random sample of size *n* and used it to evaluate a 95% confidence interval for a given index using (2). We then checked to see if the true value of the $H_{m}$ was in the interval or not. This was repeated 5000 times, and the proportion of times when the true value was in the interval was calculated. When the asymptotics works well, this proportion should be close to 0.95. We repeated this for sample sizes ranging from 10 to 1000 in increments of 10. The results for s=1.5, order m=2 and m=3 are given in Figure 1 and Figure 2; the results for s=2.5, order m=2 and m=3 are given in Figure 3 and Figure 4.

The results suggest that convergence is fast, particularly when the order is m=2. We conjecture that this may be caused by the fact that, when *m* is larger, the probabilities in the corresponding CDOTC are smaller and hence require a larger sample size for convergence. For the same reason, the results with s=1.5 converge faster than that of s=2.5, because s=2.5 yields a thinner tail distribution which requires a larger sample size for convergence. Although GSE with order m≥3 may shed some light on specific information, GSE with order m=2 is enough to well exist with asymptotic properties for any valid underlying probability distribution p.

## 4. Discussion

The proposed asymptotic properties in Corollary 1 and 2 make it possible for interval estimation and statistical tests. Based on the simulation results, the convergence is quite fast, particularly under order m=2. Note that a GSE with order m=2 already enjoys all asymptotic properties without any assumption on original distribution p.

We recommend using GSE with order m=2 in place of Shannon’s entropy in all entropy-based methods when applicable. By replacing Shannon’s entropy with GSE, one still enjoys all the benefits of Shannon’s entropy with a fast convergence speed. Moreover, using GSE is risk-free compared to Shannon’s entropy because Shannon’s entropy (1) does not exist on some thick-tailed distributions and (2) requires thinner tail distribution for some asymptotic properties [11]. Additional research is required to aid the transition. The proposed asymptotic results allow interval estimation and statistical tests on the modified entropy-based methods that replaced Shannon’s entropy with GSE. Future research should aim to provide additional estimation methods of GSE and statistical properties of functions of GSE, such as GMI. The proposed asymptotic properties in this article directly provide asymptotic normality for the plug-in estimator of GMI when the real underlying GMI is not 0. The asymptotic behavior for the plug-in estimator of GMI when the real underlying GMI is 0 remains an open question, which we will address in future work.

## 5. Proofs

The proofs require several lemmas. The first lemma is state below.

**Lemma** **1** ([11,23])**.** *Assume that $\sum_{k=1}^{\infty }p_{k}{\left|\log p_{k}\right|}^{2}<\infty$ and that there is a deterministic sequence K(n) with K(n)→∞ such that $\lim_{n\to \infty }K\left(n\right)/\sqrt{n}\to 0$ and*

$$\lim_{n\to \infty }\sqrt{n}\sum_{k=K(n)}^{\infty }p_{k}\log p_{k}=0.$$

*In this case*

$$\sqrt{n}\left({\hat{H}}_{n}-H\right)\overset{d}{\to }N\left(0,\sigma^{2}\right),$$

*where*

$$\sigma^{2}=\sum_{k=1}^{\infty }p_{k}{\left(\log p_{k}\right)}^{2}-{\left(\sum_{k=1}^{\infty }p_{k}\log p_{k}\right)}^{2}.$$

*Furthermore, if σ>0,*

$$\sqrt{n}\left(\frac{{\hat{H}}_{n}-H}{{\hat{\sigma }}_{n}}\right)\overset{d}{\to }N\left(0,1\right)$$

*where*

$${\hat{\sigma }}_{n}^{2}=\sum_{k=1}^{\infty }{\hat{p}}_{k}{\left(\log{\hat{p}}_{k}\right)}^{2}-{\left(\sum_{k=1}^{\infty }{\hat{p}}_{k}\log{\hat{p}}_{k}\right)}^{2}.$$

Different proofs of Lemma 1 are provided in [11,23].

The spirit for proof of Theorem 1 is to regard CDOTC as an original distribution and utilize the result from Lemma 1. Toward that end, several lemmas are needed and stated below.

**Lemma** **2** (Equivalent conditions in Lemma 1)**.** *For any valid distribution p, let the corresponding CDOTC with order m be denoted as $p_{m}$, then*

$$\sum_{k=1}^{\infty }p_{m,k}{\left|\log p_{m,k}\right|}^{2}<\infty$$

*and that there is a deterministic sequence K(n) with K(n)→∞ such that $\lim_{n\to \infty }K\left(n\right)/\sqrt{n}\to 0$ and*

$$\lim_{n\to \infty }\sqrt{n}\sum_{k=K(n)}^{\infty }p_{m,k}\log p_{m,k}=0.$$

**Lemma** **3** ($\sigma_{m}^{2}$ in Theorem 1)**.** *In Theorem 1,*

$$\sigma_{m}^{2}=\sum_{k=1}^{\infty }{\left[\frac{m^{2}}{p_{k}}\left(p_{m,k}\ln p_{m,k}+p_{m,k}H_{m}\left(Z\right)\right)\right]}^{2}.$$

**Lemma** **4** (${\hat{\sigma }}_{m}^{2}$ in Corollary 1)**.** *In Corollary 1,*

$${\hat{\sigma }}_{m}^{2}=\sum_{k=1}^{\infty }{\left[\frac{m^{2}}{{\hat{p}}_{k}}\left({\hat{p}}_{m,k}\ln{\hat{p}}_{m,k}+{\hat{p}}_{m,k}{\hat{H}}_{m}\left(Z\right)\right)\right]}^{2}.$$

**Proof of** **Lemma 2.** Note that for any p to be a valid distribution, the tail of p must be thicker than 1/(klnk) because $\sum_{k\geq 2}1/\left(k\ln k\right)$ diverges. Hence $p_{m}$ is thicker than $1/(k^{2}\ln^{2}k)$ for any m≥2 by definition. It is shown in Example 3 of [11] that such tail satisfies the mentioned conditions. □

**Proof** **of Lemma 3.** Because of Lemma 2, $\sigma^{2}$ can be obtained under finite *K* and then let K→∞. For a finite *K*, it can be verified that for i=1,…,K−1,

$$\frac{\partial H_{m}}{\partial p_{i}}=\left(\ln p_{m,K}-\ln p_{m,i}\right)\frac{mp_{m,i}}{p_{i}}-m\left(\frac{p_{m,i}}{p_{i}}-\frac{p_{m,K}}{p_{K}}\right)\left(H_{m}+\ln p_{m,K}\right).$$

Let

$$\begin{array}{cc} & v={\left(p_{1},\ldots ,p_{K-1}\right)}^{\tau },\\ & \hat{v}={\left({\hat{p}}_{1},\ldots ,{\hat{p}}_{K-1}\right)}^{\tau }. \end{array}$$

We have $\sqrt{n}\left(\hat{v}-v\right)\overset{L}{\to }MVN\left(0,\Sigma \left(v\right)\right)$, where Σ(v) is the (K−1)×(K−1) covariance matrix given by

$$\Sigma \left(v\right)=\left(\begin{array}{cccc} p_{1}\left(1-p_{1}\right) & -p_{1}p_{2} & \ldots & -p_{1}p_{K-1}\\ -p_{2}p_{1} & p_{2}\left(1-p_{2}\right) & \ldots & -p_{2}p_{K-1}\\ \ldots & \ldots & \ldots & \ldots \\ -p_{K-1}p_{1} & -p_{K-1}p_{2} & \ldots & p_{K-1}\left(1-p_{K-1}\right) \end{array}\right)$$

According to the first-order Delta method,

$$\sigma_{K}^{2}=\nabla H_{m}^{T}\Sigma \nabla H_{m}=\sum_{k=1}^{K}{\left[\frac{m^{2}}{p_{k}}\left(p_{m,k}\ln p_{m,k}+p_{m,k}H_{m}\left(Z\right)\right)\right]}^{2}.$$

Given Lemma 2, let K→∞,

$$\sigma^{2}=\sum_{k=1}^{\infty }{\left[\frac{m^{2}}{p_{k}}\left(p_{m,k}\ln p_{m,k}+p_{m,k}H_{m}\left(Z\right)\right)\right]}^{2}.$$

 □

**Proof of** **Lemma 4.** Lemma 4 is because of ${\hat{\sigma }}_{m}^{2}\overset{p}{\to }\sigma_{m}^{2}$. □

**Proof of Theorem 1 and Corollary** **1.** With Lemmas 1–4, and Slutsky’s theorem, Theorem 1 and Corollary 1 are proved. □

**Proof of Corollary** **2.** Corollary 2 is a directly result of Theorem 1, except under uniform distribution when $\nabla H_{m}=0$ for all m≥2. □

## Abbreviations

The following abbreviations are used in this manuscript:

CDOTC	Conditional Distribution of Total Collision
GMI	Generalized Mutual Information
GSE	Generalized Shannon’s Entropy
ML	Machine Learning
